# Patient Acceptance of Rapid HIV Testing During Targeted Screening in the Emergency Department

**DOI:** 10.5811/westjem.48500

**Published:** 2025-07-18

**Authors:** Brianna N. McMonagle, Robert Braun, Jude Luke, Anita Goel, Caroline Freiermuth

**Affiliations:** * University of Cincinnati College of Medicine, Cincinnati, Ohio; † University of Cincinnati Medical Center, Department of Emergency Medicine, Cincinnati, Ohio

## Introduction

It is concerning that 12.8% of the 1.2 million individuals in the U.S. living with HIV are undiagnosed. It is important to identify these patients so those affected receive evidence-based treatment and prevent spread. The objective of this study is to estimate acceptance rates of free, point of care fingerstick HIV testing for a sample of targeted, at-risk individuals in the emergency department (ED). Additionally, we assess how test acceptance varies with demographics and categorize why patients decline HIV testing.

## Methods

This is a single-center retrospective analysis of survey responses and documented HIV testing in a targeted sample of patients presenting to an urban academic ED in the Midwest from 2022–2023. The survey and testing were done by trained health promotion advocates, who perform targeted screening on a convenience sample of patients with social risk factors. We report the prevalence of testing acceptance and reasons for declining testing. Comparison of demographics between the overall ED population and those offered testing was done using a two sample T-test for age, and Chi-squared testing for all other variables. Logistic regression was done to find associations between test acceptance and demographic or risk factors.

## Results

Over 24-months, 3,249 unique patients were offered point-of-care (POC) HIV tests and 1,680 (51.7%) accepted, per Table 1. African American patients and those identifying as males were offered testing at a greater frequency than their overall representation in the ED. African American patients were more likely to accept testing than white patients (54.3% vs. 47.8%, OR=1.28, p< 0.001), while increased age was associated with decreased test acceptance (OR=0.98 per year, p< 0.001), per Table 2. All patients who accepted belong to at least one population of interest including high-risk heterosexual behavior (40.4%), youth ages 13–29 (34.9%), and women of color (25.8%). The most common reasons cited by patients who declined testing were not wanting to be interviewed (24%) and having a prior negative test within the last 3 months (19.1%).

## Conclusions

Over half of those offered POC testing for HIV in the ED accepted, with a significant percentage of those who declined reporting recent negative testing. Younger age and African American ethnicity were associated with a higher acceptance rate. Adopting an opt-out screening system and addressing common reasons for declining testing may provide opportunities for increasing HIV test uptake.

## Figures and Tables

**Table 1:** Characteristics of Emergency Department Patients and those offered rapid POC HIV testing, by acceptance

	ED Patients (n = 65,215)	Offered (n = 3,249)	Accepted (n = 1,680)	Declined (n = 1,569)
Demographics				
Age	42 (29–60)	35 (27–47)	34 (26–44)	37 (28–50)
Race				
Black/African American	25,836 (39.6)	1,655 (50.9)	899 (53.5)	756 (48.2)
White	31,196 (47.8)	1,413 (43.5)	676 (40.2)	737 (47.0)
Hispanic	5,184 (7.9)	79 (2.4)	49 (2.9)	30 (1.9)
American Indian/Alaska native	115 (0.2)	7 (0.2)	4 (0.2)	3 (0.2)
Asian/Pacific Islander	775 (1.2)	18 (0.6)	10 (0.6)	8 (0.5)
Biracial/Multicultural	633 (1.0)	42 (1.3)	25 (1.5)	17 (1.1)
Other/Unknown	1,476 (2.3)	35 (1.1)	17 (1.0)	18 (1.1)
Sex (at birth)				
Male	32,793 (50.3)	1,880 (57.9)	936 (55.7)	944 (60.2)
Female	32,377 (49.6)	1,369 (42.1)	744 (44.3)	625 (39.8)
Unknown	45 (0.1)	0 (0.0)	0 (0.0)	0 (0.0)
Gender Identity				
Male	32,685 (50.1)	1,872 (57.6)	931 (55.4)	941 (60.0)
Female	32,239 (49.4)	1,362 (41.9)	740 (44.0)	622 (39.6)
Transgender/Nonbinary	252 (0.4)	15 (0.5)	9 (0.5)	6 (0.4)
Unknown	39 (0.1)	0 (0.0)	0 (0.0)	0 (0.0)
Insurance Status				
None		368 (11.3)	206 (12.3)	162 (10.3)
Medicaid		2,141 (65.9)	1,117 (66.5)	1,024 (65.3)
Medicare		135 (4.2)	57 (3.4)	78 (5.0)
Dual Medicaid/Medicare		101 (3.1)	49 (2.9)	52 (3.3)
Private		278 (8.6)	149 (8.9)	129 (8.2)
Military/VA		6 (0.2)	3 (0.2)	3 (0.2)
Other/Unknown		220 (6.8)	99 (5.9)	121 (7.7)
Primary Care Status				
Has primary care		1,355 (41.7)	739 (44.0)	616 (39.3)
No primary care		1,233 (38.0)	736 (43.8)	497 (31.7)
Not Assessed/Unknown		661 (20.3)	205 (12.2)	456 (29.1)
Has primary care		1,355 (41.7)	739 (44.0)	616 (39.3)
No primary care		1,233 (38.0)	736 (43.8)	497 (31.7)
Not Assessed/Unknown		661 (20.3)	205 (12.2)	456 (29.1)

Age is reported as median (IQR), all other variables are n (%).

*POC*, point-of-care; *HIV, h*uman immunodeficiency virus; *VA*, Veterans Affairs.

**Table 2:** Logistic regression model for Emergency Department patients’ acceptance of rapid POC HIV testing

	OR	95% CI	p-value
Demographics			
Age, per year	0.98	0.98–0.99	<0.001
Race			
White	^*^Reference		
Black/African American	1.28	1.17–1.53	<0.001
Hispanic	1.54	0.74–1.62	0.07
Other/Unknown	1.17	0.56–1.32	0.46
Sex (at birth)			
Male	^*^Reference		
Female	1.11	0.95–1.24	0.17
Insurance Status			
None	^*^Reference		
Medicaid	0.90	0.85–1.28	0.36
Medicare	0.73	0.53–1.16	0.13
Dual Medicaid/Medicare	0.92	0.60–1.38	0.73
Private	0.95	0.80–1.46	0.74
Other/Unknown	0.66	0.43–0.80	0.02

*OR*, odds ratio; *CI*, confidence interval.

## References

[1] Center for Disease Control and Prevention (2024). HIV Surveillance Supplemental Report: Estimated HIV Incidence and Prevalence in the United States, 2018–2022.

